# A cross sectional assessment of knowledge, attitude and practice towards Hepatitis B among healthy population of Quetta, Pakistan

**DOI:** 10.1186/1471-2458-12-692

**Published:** 2012-08-23

**Authors:** Noman ul Haq, Mohamed Azmi Hassali, Asrul A Shafie, Fahad Saleem, Maryam Farooqui, Hisham Aljadhey

**Affiliations:** 1 Department of Pharmacy, University of Baluchistan, Quetta, Pakistan; 2 Discipline of Social and Administrative Pharmacy, School of Pharmaceutical Sciences, Universiti Sains Malaysia, Penang, Malaysia; 3 Discipline of Social and Administrative Pharmacy, School of Pharmaceutical Sciences, Universiti Sains Malaysia, Penang, Malaysia; 4 Faculty of Pharmacy, Universiti Teknologi MARA (UiTM), Bertam, Penang, 13200, Malaysia; 5 College of Pharmacy, King Saud University, Riyadh, Saudi Arabia

**Keywords:** Knowledge, Attitude, Practice, Hepatitis B, Healthy population, Pakistan

## Abstract

**Background:**

Hepatitis B (HB) is a serious global public health problem. This study aims to evaluate Knowledge, Attitude and Practice (KAP) towards Hepatitis B (HB) among healthy population of Quetta city, Pakistan.

**Methods:**

A cross sectional, descriptive study was undertaken. One thousand healthy individuals (aged 18 years and above) were approached for the study. KAP towards HB was assessed by using a pre validated questionnaire. Descriptive statistics were used for elaborating patients’ demographic characteristics. Inferential statistics (Mann–Whitney *U* test and Kruskal Wallis test) were used for comparison while Spearman’s rho correlation was used to identify association between the study variables. All analyses were performed using SPSS 16.0.

**Results:**

Out of 1000 distributed questionnaires, 780 were returned with a response rate of 78.0%. Four hundred and twenty (53.8%) respondents were male with mean age of 32.76 ± 9.40 year. Two hundred and eight (26.7%) had intermediate level of education and 354 (45.4%) were unemployed. Mean scores for knowledge, attitude and practice were 8.74 ± 2.7, 3.72 ± 1.2 and 2.76 ± 1.1 respectively. Significant and positive linear correlations between knowledge-attitude (r = 0.296, p < 0.01) knowledge-practice (r = 0.324, p < 0.01) and attitude-practice (r = 0.331, p < 0.01) were observed. Area of residence (locality) was the only variables significantly associated with mean KAP of the study respondents.

**Conclusion:**

Results from the current study heighted poor KAP of healthy population towards HB. The positive linear correlations reaffirms that better knowledge can lead to positive attitude and subsequently in good practices. This will further help in prevention and management of HB. Therefore, extensive health educational campaign should be provided to general population and especially to the residents of rural areas.

## Background

Hepatitis B (HB) is a serious and common infectious disease of the liver. The World Health Organization (WHO) in 2009 reported HB to infect nearly 2 billion people around the globe. Furthermore, out of those 2 billion, 350 million suffered from chronic, lifelong infection [1]. Moreover, an estimated 15–40% of chronic HB carriers were susceptible to develop liver cirrhosis and hepatocellular carcinoma [2,3]. HB is a confronting ailment and results in 0.6 million deaths annually [4,5]. Although HB is classified as ‘*disease of priority,’* there is an incessant increase in detection of new cases worldwide [6]. Furthermore, HB is widespread in the Asia Pacific region and 10 to 15 million of the population suffer from this disease [7-9]. Similar to what is reported worldwide, the incidence of HB infection is on continuous rise in Pakistan [10]. The pervasiveness of HB ranges between 7 to 20% in Pakistani population and varies from region to region [9,11-15]. In metropolitan cities like Karachi, the prevalence of HB is reported lower (2-5%) as compared with the rural areas which can range from where 30-35% [11,13,15]. Based on these statistics, Pakistan is classified as a region of ‘*intermediate risk’* towards HB by WHO [1].

The health system of Pakistan consists of both private and public sectors. The private health sector serves nearly 70% of the population, whereas the public sector comprises more than 10,000 health facilities, ranging from basic health units (BHUs) to tertiary referral centers [16]. Within this context, Quetta is the largest city and the provincial capital of the Balochistan Province of Pakistan. The city is located near the Durand Line border with Afghanistan and is the only city in with adequate health institutes and facilities. The area holds the attraction for treatment of acute and chronic diseases both for the local population as well as immigrants from Afghanistan and Iran.

Knowledge, attitude, and practice (KAP) surveys are representative of a specific population to collect information on what is known, believed and done in relation to a particular topic, and are the most frequently used study tool in health-seeking behavior research [17]. Knowledge is usually assessed in order to see how far community knowledge corresponds to biomedical concepts [18]. Typical questions include knowledge about causes and symptoms of the illness under investigation. People reported knowledge which deviates from biomedical concepts is usually termed as ‘beliefs’ [19]. Attitude has been defined as “a learned predisposition to think, feel and act in a particular way towards a given object or class of objects” [20]. As such, attitude is a product of a complex interaction of beliefs, feelings, and values. Practices in KAP surveys usually enquire about the use of preventive measures or different health care options. Normally, hypothetical questions are asked, therefore it hardly permits statements about actual practices, rather, it yields information on people’s behaviors or on what they know should be done [21].

As discussed earlier, the frequency of HB is increasing progressively worldwide, prevention is considered as one best way to safeguard populations’ health. Deterrence can also lead to decreased spread of HB virus thus reducing the chances of disease conduction. Prevention against any disease is proportional to KAP of the population and is reflective of the importance that is paid to health related issues by the society. Therefore, KAP studies play an imperative role in determining the ambiguities of a society and are widely used in population reported assessment research worldwide. To the contrary, there is paucity of data from Pakistan and KAP towards HB among healthy population is never explored. In spite of the efforts made by authorities to raise knowledge and awareness about HB, no progress is reported. In the present scenario, there is a need to assess the KAP status of healthy individuals towards HB so that the information can be used to develop a better and need based program for the society. The current study aimed to assess knowledge, attitudes and practices (KAP) of healthy population towards HB from Quetta city, Pakistan.

## Methods

### Study design and settings

This descriptive study was designed as questionnaire based, cross sectional analysis. Healthy population from Quetta city, Pakistan was targeted for the study. The study questionnaire was distributed at places of common interest (shopping malls, educational institutes, households etc.). Participants were requested to answer the questionnaire on spot and were subsequently collected after completion.

### Study sample

By employing convenience sampling method, 1000 respondents were targeted for the study. The study was conducted from April 2011 to July 2011. Healthy individuals aging 18 years and above, with no physical and mental mutilation, not using any type of medication, and familiarity with Urdu (National language of Pakistan) were included in the study. Patients having reported illness and immigrants from other countries were excluded from data collection.

### Ethical approval

This study was performed according to the ethical standards for human experimentation [22]. The Joint Clinical Research Committee approved the study protocol (No: EA/NUH/1205-2009). Written consent was also taken from the respondents before initiation of the research.

### Study instrument

A self administered, 35 itemed questionnaire comprising of four sections was used for data collection. In addition to the demographic data, 20 questions explored knowledge towards HB, 7 questions focused on attitude and 8 questions addressed practices towards HB. Respondents were asked to answer in limited as well as multiple choice formats. The primary version of the questionnaire was developed through extensive literature review in English language [17,23]. It was later translated into Urdu language by using standard translating procedures [24,25]. The Urdu version of the questionnaire was tested for its reliability and validity. Internal consistency was assessed by using Cronbach’s alpha (α = 0.7) and was found to be in acceptable ranges [26]. Face, content and convergent validity of the questionnaire was performed by experts at Discipline of Social and Administrative Pharmacy, School of Pharmaceutical Sciences, Universiti Sains Malaysia. The questionnaire was than piloted with 30 respondents for its acceptability and consistency. Little modification was needed after the pilot testing. Data from the pilot study was not included in the final analysis. As the consistency and validity of the study questionnaire was stabilized, the instrument was made available for data collection.

### Statistical analysis

Descriptive statistics were used to illustrate respondents’ demographic characteristics. Categorical variables were measured as percentages while continuous variables were expressed as mean ± standard deviation. The Kolmogrov-Smirnov test was applied to declare the nature of data distribution. Inferential statistics (Mann–Whitney *U* test and Kruskal Wallis tests) were used to assess the difference while Spearman's rank correlation coefficient was used to evaluate the relationship between the study variables. A p value of <0.05 was taken as significant for Mann–Whitney *U* test and Kruskal Wallis test. In addition, p < 0.01 was taken as significant for correlation analysis. Statistical Package for Social Sciences (SPSS) v. 16.0 was sued for data analysis.

## Results

### Demographic characteristics

A total of 1000 questionnaires were distributed and 780 were received with a response rate of 78.0% as shown in Table 1. The Kolmogrov-Smirnov test revealed non normal distribution of the data. The gender distribution was almost equal with 417 (53.5%) of males. Mean age of the study participants was 32.7 ± 9.40 years with 428 (54.9%) having urban residency. One hundred and ninety eight (25.4%) had intermediate level of education and 364 (46.7%) were unemployed (including house wives and students). One hundred and twenty two (15.6%) had monthly income of Pakistan Rupees (Pk. Rs) of in between 5001-10,000. The major source of information regarding HB was from family/friend/neighbors 284 (36.4%), followed by 138 (17.7%) by HB information leaflets/brochures and posters.

**Table 1:** Characteristics of the study respondents (N = 780)

**Characteristics**	**N**	**%**
***Age (32.61 ± 9.48)***		
18-27	283	36.3
28-37	268	34.4
38-47	171	21.9
48-57	58	7.4
***Gender***		
Male	417	53.5
Female	363	46.5
***Education***		
Illiterate	35	4.5
Religious Only	58	7.4
Primary	185	23.7
Metric	79	10.1
Intermediate	198	25.4
Graduation	144	18.5
Post-Graduation	81	10.4
***Occupation***		
Unemployed	364	46.7
Government Servant	134	17.2
Private Servant	189	24.2
Self Employed	93	11.9
Income*		
No Income	366	46.9
< Pk. Rs. 5000	108	13.8
5001-10000	122	15.6
10001-15000	91	11.7
>15001	93	11.9
***Locality***	428	54.9
Urban	352	45.1
Rural		
***Source of HB information***		
New papers and magazines	138	17.4
Health workers	100	12.8
Family/friends/neighbors	284	36.4
TV, Radio and Internet	114	14.9
Religious leaders/teachers	6	0.7
HB information leaflets, brochures, posters etc.	138	17.7

*1 Pk Rs (Pakistani rupees) = 0.01172 USD (US dollars).

### Assessment of knowledge towards Hepatitis B

Table 2 describes the responses of the participants towards HB knowledge. Knowledge was assessed by questions focusing on HB etiology, sign and symptoms, transmission, treatment and management. Each response was scored as ‘yes’ or ‘no’. The scoring range of the questionnaire was 20 (maximum) to 0 (minimum). A cut off level of ≤ 11 was considered as poor whereas > 11 was considered as adequate knowledge about HB. Knowledge scores for individuals were calculated and summed up to give the total knowledge score.

**Table 2:** Responses to Hepatitis B knowledge items

**Hepatitis B Knowledge Items**	**Yes**	**No**
	**N (%)**	**N (%)**
Have you ever heard of a disease termed as Hepatitis?	763 (97.8)	17 (2.2)
Have you ever heard of a disease termed as Hepatitis B?	655 (84.0)	125 (16)
Is Hepatitis B is a viral diseases?	357 (45.8)	423 (54.2)
Can Hepatitis B affect liver function?	449 (57.6)	331 (42.4)
Can Hepatitis B cause liver Cancer?	218 (27.9)	562 (72.1)
Can Hepatitis B affect any age group?	211 (27.1)	569 (78.9)
The early symptoms of Hepatitis B are same like cold and flu (fever, running nose, cough)	138 (17.7)	642 (82.3)
Jaundice is one of the common symptoms of Hepatitis B?	252 (32.3)	528 (67.7)
Are nausea, vomiting and loss of appetite common symptom of Hepatitis B?	163 (20.9)	617 (79.1)
Are there no symptoms of the Hepatitis B in some of the patients?	145 (18.6)	635 (81.4)
Can Hepatitis B be transmitted by un-sterilized syringes, needles and surgical instruments?	265 (34.0)	515 (66.1)
Can Hepatitis B be transmitted by contaminated blood and blood products?	269 (37.9)	511 (62.1)
Can Hepatitis B be transmitted by using blades of the barber/ear and nose piercing?	260 (33.3)	520 (66.7)
Can Hepatitis B be transmitted by unsafe sex?	141 (10.1)	639 (81.9)
Can Hepatitis B be transmitted from mother to child?	187 (24.0)	593 (76.0)
Can Hepatitis B be transmitted by contaminated water/food prepared by person suffering with these infections?	242 (31.0)	538 (68.5)
Is Hepatitis B curable/treatable?	458 (58.7)	322 (41.3)
Can Hepatitis B be self-cured by body?	375 (48.1)	405 (51.9)
Is vaccination available for Hepatitis B?	598 (76.7)	182 (23.3)
Is specific diet is required for the treatment of Hepatitis B?	649 (83.2)	131 (16.8)

Note: Knowledge was assessed by giving 1 to correct answer and 0 to the wrong answer. The scale measured knowledge from maximum 20 to minimum 0. Scores < 11 were taken as poor, ≥ 11 as adequate knowledge of Hepatitis B. Mean knowledge was 8.74 ± 2.7.

Out of the 780 participants, 588 (75.4%) were within the poor knowledge range whereas 192 (24.6%) showed adequate knowledge about HB. Poor knowledge was apparent in responses to questions relating to symptoms (question 7-10) and transmission of HB (question 11-16). Correct answers to these questions were 17.7, 32.3, 20.9 and 18.6% for symptoms while 34.0, 37.9, 33.3, 10.1, 24.0, and 31.0% for transmission of HB respectively. Correct answers, 97.8, 84.0, 76.7 and 83.2% were highest in response to questions 1,2,19 and 20 respectively. The mean knowledge score for the entire study cohort was 8.74 ± 2.7.

### Assessment of attitude towards Hepatitis B

Attitude towards HB was assessed by asking seven questions as shown in Table 3. Each question was labeled with positive or negative attitude. A score of 1 was given to positive while 0 was given to negative attitudes with a score range of maximum of 7 to a minimum of 0. The scale classified attitude as positive with score > 4 and negative ≤ 4. Majority of the respondent 622 (79.7%) believed that they can never get infected with HB. Three hundred and eight (39.5%) respondents stated that they will be ashamed to get infected with HB. Five hundred and forty nine (70.4%) of the study respondents were of opinion to use complementary and alternative system in case of HB infection. In addition, 203 (26.0%) agreed to consult a physician as their first choice of treatment. However, respondents were ready to disclose their disease to their spouse (n = 367, 47.1%) and parents (n = 207, 26.5%). Over all the respondents had a negative attitude towards Hepatitis B with mean score of 3.72 ± 1.2.

**Table 3:** Attitude toward Hepatitis B

**Hepatitis B Attitude Items**	**N**	**%**
***Do you think you can get Hepatitis B?***		
Yes*	158	20.3
No	622	79.7
***What would be your reaction if you found that you have Hepatitis B?***		
Fear*	246	31.5
Shame	308	39.5
Surprise	89	11.4
Sadness	137	17.6
***Who would you talk to about your illness?***		
Physician	104	13.3
Spouse	367	47.1
Parents	207	26.5
Child	7	0.9
Other Relatives	23	2.9
Friends	72	9.2
No one^¥^	0	0
***What will you do if you think that you have symptoms of Hepatitis B?***		
Go to Health facility*	231	29.6
Go to Hakeem	273	35.0
Go to Homeopath	73	9.4
Go to Traditional healer	203	26.1
***If you had symptoms of Hepatitis B, at what stage you will go to the health facility?***		
Own treatment fails	405	51.9
After 3-4 weeks of the appearance of symptoms	126	16.2
Soon as I realize the symptoms are of Hepatitis B*	203	26.0
Will not go to physician	46	5.9
***How expensive do you think is the diagnosis and treatment of Hepatitis B?***		
Free	5	0.6
Reasonable	51	6.5
Somewhat expensive	338	43.3
Expensive	248	31.8
Don't know^¥^	138	17.7
***What worries you most if you will be diagnosed with Hepatitis B***		
Fear of death	259	32.2
Fear of disease spread to family	149	19.1
Cost of treatment	81	10.4
Isolation from the society^¥^	291	37.3

^*^Positive attitude,^*¥*^*Negative attitude.*

*Note: Attitude was assessed by giving 1 to positive and 0 to negative attitude. The scale classified attitude as positive with score >4 and negative ≤4. Over all the respondents had a negative attitude towards Hepatitis B with mean score of 3.72 ± 1.2*.

### Assessment of practices towards Hepatitis B

Practices towards HB were assessed by asking eight questions as shown in Table 4. Each question was labeled with good or poor practice. A score of 1 was given to good while 0 was given to bad practice with a score range of maximum of 8 to a minimum of 0. The scale classified practice as good with score > 5 and poor ≤ 5. Majority of the respondents, 756 (96.9%) never went for HB screening and 674 (86.8%) stated a negative immunized status against HB. It was interesting to know that 634 (81.8%) never asked for a new syringe when required, while only 344 (44.1%) agreed with the statement that they ask for screening of blood and blood products before transfusion. Six hundred and thirty one (80.9%) respondents either never asked the barber to use new blade, or for safe and clean equipment for nose and ear piercing. However, on the contrary majority of the study participants (n = 635, 81.4%) revealed that they avoid meeting a person infected with HB. Majority 731 (93.7%) agreed that they will go for further investigation and treatment if they are infected with HB. In addition, a small number of respondents (n = 14, 1.8) have ever attended any educational program on HB. The mean score for HB related practices was 2.76 ± 1.1 revealing poor practices among the study participants.

**Table 4:** Practice related to Hepatitis B

**Hepatitis B Practice Items**	**Yes**	**No**
	**N (%)**	**N (%)**
Have you done screening for Hepatitis B?	24 (3.1)	756 (96.9)
Have you got yourself vaccinated against Hepatitis B?	103 (13.2)	674 (86.8)
Do you ask for a new syringe before use?	146 (18.7)	634 (81.3)
Do you ask for screening of blood before transfusion?	344 (44.1)	436 (55.9)
Do you ask your barber to change blade/Or for safe equipments for ear and nose piercing?	149 (19.1)	631 (80.9)
In case you are diagnosed with Hepatitis B, would you go for further investigation and treatment?	731 (93.7)	49 (6.3)
Do you avoid meeting Hepatitis B patients?	635 (81.4)	145 (18.6)
Have you ever participated in health education program related to Hepatitis B?	14 (1.8)	766 (98.2)

Note: Practice was assessed by giving 1 to positive and 0 to negative attitude. The scale classified practice as good with score >5 and poor ≤5. Over all the respondents reported to have poor practice towards Hepatitis B with mean score of 2.76 ± 1.1.

### Association of demographic characteristics and mean KAP Scores

The association of demographic characteristics and mean KAP scores is presented in Table 5. Among the demographic variables, only area of residence (locality) was significantly associated with mean KAP scores (p < 0.01).

**Table 5:** Comparison of Demographic Characteristics and Mean KAP Scores

**Description**	**N (780)**	**Knowledge Score (Mean ± SD)**	**P-Value**	**Attitude Score (Mean ± SD)**	**P-Value**	**Practice Score (Mean ± SD)**	**P-Value**
Age*							
18-27	283	8.98 (2.6)	0.237	3.73 (1.2)	0.075	2.66 (0.9)	0.071
28-37	268	8.76 (2.6)		3.67 (1.3)		2.76 (1.0)	
38-47	171	8.37 (2.8)		3.91 (1.3)		3.05 (1.5)	
48-57	58	8.48 (2.8)		3.40 (1.1)		2.29 (1.1)	
Gender**							
Male	417	8.83 (2.5)	0.537	3.72 (1.3)	0.197	2.83 (1.3)	0.747
Female	363	8.62 (2.8)		3.72 (1.2)		2.66 (0.9)	
Education*							
Illiterate	35	4.31 (1.6)	0.105	3.09 (1.0)	0.107	2.09 (0.8)	0.155
Religious Only	58	7.97 (2.6)		3.50 (0.9)		2.47 (0.9)	
Primary	185	7.62 (2.4)		3.28 (1.1)		2.21 (0.7)	
Metric	79	7.70 (2.3)		3.49 (1.0)		2.80 (0.9)	
Intermediate	198	9.35 (1.8)		3.76 (1.2)		2.72 (0.8)	
Graduation	144	10.08 (2.3)		3.98 (1.4)		3.15 (1.1)	
Post-Graduation	81	10.89 (2.3)		4.85 (1.2)		3.84 (1.7)	
Occupation*							
Unemployed	364	8.22 (2.7)	0.231	3.55 (1.1)	0.224	2.63 (0.9)	0.085
Government Servant	134	9.54 (2.5)		3.86 (1.2)		3.20 (1.4)	
Private Servant	189	9.13 (2.6)		4.05 (1.4)		2.65 (1.1)	
Self Employed	93	8.78 (2.3)		3.56 (1.3)		2.82 (1.4)	
Income*							
No Income	366	8.27 (2.8)	0.095	3.54 (1.1)	0.412	2.62 (0.9)	0.221
< Pak Rs. 5000	108	8.25 (2.9)		3.81 (1.5)		2.26 (0.9)	
5001-10000	122	8.87 (2.3)		3.41 (1.3)		2.28 (0.8)	
10001-15000	91	9.44 (2.6)		4.01 (1.2)		3.07 (0.8)	
>15001	93	10.26 (1.6)		4.49 (1.1)		4.18 (1.4)	
Locality**							
Urban	428	9.34 (2.4)	***0.001***	3.98 (1.3)	***0.001***	3.05 (1.2)	***0.001***
Rural	352	8.00 (2.8)		3.42 (1.1)		2.40 (0.8)	
**Total**	**780**	**8.74 (2.7)**		**3.72 (1.2)**		**2.76 (1.1)**	

* Kruskal Wallis Test, ** Mann Whitney Test, p < 0.05.

### Correlation between knowledge, attitude and practice

Correlations were interpreted using the following criteria: 0–0.25 = weak correlation, 0.25–0.5 = fair correlation, 0.5–0.75 = good correlation and greater than 0.75 = excellent correlation [27]. The correlation revealed significant positive linear correlations between knowledge-attitude (r = 0.296, p < 0.01) knowledge-practice (r = 0.324, p < 0.01) and attitude-practice (r = 0.331, p < 0.01). The result reaffirms the relationship between knowledge attitude and practice with infection control measures as shown in Table 6. 

**Table 6:** Correlation between knowledge, attitude, and practice scores

**Variable**	**Correlation coefficient**	P-Value*
Knowledge-Attitude	0.296	<0.01
Knowledge-Practice	0.324	<0.01
Attitude-Practice	0.331	<0.01

*Correlation significant at 0.01 level (2 tailed).

## Discussion

The current study sought to evaluate KAP towards HB among healthy individuals. Results of the study revealed poor KAP towards HB. The mean knowledge score was 8.74 ± 2.7 indicating low level of knowledge towards HB among the study cohort. A small percentage of respondents actually knew about transmission of HB. Lack of knowledge about HB transmission can be attributed to rise in the frequency of HB. Only 28.2% of the participant believed that HB can cause liver cancer, which is again a major sign of concern. The primary source of information was through family, friends and neighbors. These results are in line with the findings from studies reported from other parts of Pakistan where the overall knowledge of the general population regarding HB was reported low [28,29]. Similar to what is reported from Pakistan, poor knowledge regarding HB also is also reported around the globe [30-37]. On the contrary Shalaby et al. (2007) in Egypt reported that participants had adequate knowledge towards transmission, vaccination and treatment of HB [38]. Possible reasons that can be attributed to this difference of response are demographic variation of the study population, study location and as well as the study tool used for data collection.

In addition, mean attitude score was also found lower in the study participants. Majority of the participants reported to use complementary and alternative medicines if infected with HB. Home remedies, herbal and traditional therapies were the treatment of choice until there is no improvement in the sign and symptoms of HB. Consulting the physicians was sought as the last resort, when all other healing system fails to provide cure. This is similar to what is reported by a study of same nature in Pakistan [28]. It is imperative to account that delay in seeking medical treatment for the infection results in further deterioration of the condition and can cause spread of infections.

An important feature of patient care revolves around the Health Belief Model which highlights individuals’ attitudes and beliefs responsible for particular health behaviour [39]. In addition, perceived benefits and barriers in the health care regimen play a vital role in achieving therapeutic success. Results from the current study revealed that general population is in habit of making independent assessments of their current health status and were favoring different health systems (spiritual healers, yunani, ayurvedic and homeopathic healing systems) resulting in medical pluralism. Multiple opinions from such entities can complicate and disseminate irrational information and practices towards HB in the population. Although some of the systems are not approved by the official authorities in Pakistan, their influence is pronounced in the population. The results notwithstanding, it is important that the general population should be educated on all aspects of HB rather than on a single or a few issues. It should also be addressed by social and medical researchers foregoing achieve a fuller understanding of the underlying issues while the outcome of such studies should be utilized in policy and decision-making by government officials and members of the health care team.

Within this context, poverty, cultural beliefs and perceived severity of illness can be the reasons of seeking alternative methods of treatment. Though not infected yet, majority of the participants perceived HB treatment as costly. This could be due to their experiences with friends and family members having difficulties in bearing the cost of HB treatment. Cost of treatment is another factor which forces the patients to seek help from traditional healers. In developing countries like Pakistan, access to the traditional healers is economical than seeking treatment at medical health care facilities.

Participants of the current study showed poor practice towards HB. Majority of the participants were not concerned about the safety measures which defiantly expose them to the danger of acquiring HB infection. Despite having awareness regarding the availability of HB vaccines, majority of the participants were not immunized against HB. Similar results were reported by Razi and colleagues in 2010 from Pakistan [40] and Kabir et al. in 2010 from Iran [41] where the participants reported to have poor practices which were directly related to the knowledge and awareness regarding HB infection. On the contrary, Shalaby et al. (2007) in Egypt reported the that the participants have good practice regarding hepatitis B hence have lower prevalence of infection [38]. Furthermore, the knowledge among participants was reported adequate by Shalaby et al., which is proportionately related to the attitude and practices of the participants.

Area of residence (locality) was the only significant factor associated the mean KAP scores. However, through extensive literature review, no studies were found to report the relation of locality and mean KAP scores. Cheung et al. in 2005 and Wu et al. in 2007 however reported education level as the significant factor associated with KAP scores of their study participants [32,42].

The positive correlations between knowledge-attitude, knowledge-practice and attitude-practice in this study reaffirm the relationship between knowledge attitude and practice with infection control measures. It is concluded that adequate knowledge can lead to positive attitude resulting in good practices. The findings are in line with the results presented by Singh et al. in 2010 [43].

## Conclusion

Summarizing the results of this study, these findings indicate a lack of understanding of the basics of infection control and the prevention of transmission of HB. Extensive health education campaign should be provided to general population and especially to the residents of rural areas. We recommend the adaptation of collaborative care where physicians, pharmacists and nurses should play their role in providing HB education to the society. Empowering the people by providing them ample education and targeting at least one member of each family to have adequate information about HB can help in managing and controlling the infection.

### Limitations

The study was conducted in one city and therefore results of the research are not representative of the entire population of Pakistan.

## Competing interests

The authors have declared no competing interests. No funding was received for this study.

## Authors’ contribution

NUH and FS conducted the survey and drafted the initial manuscript. MAH and AAS designed and supervised the study. MF and HA helped in statistical analysis, interpretation and manuscript revision. All authors read and approved the final manuscript.

## Pre-publication history

The pre-publication history for this paper can be accessed here:

http://www.biomedcentral.com/1471-2458/12/692/prepub
